# Virtual Community Outreach Program during the pandemic of COVID‐19

**DOI:** 10.1002/jgf2.424

**Published:** 2021-02-17

**Authors:** Kento Sonoda

**Affiliations:** ^1^ Department of Family Medicine University of Pittsburgh Medical Center Shadyside Pittsburgh PA USA

To the Editor,

Many Japanese people in the United States struggle with seeking medical care because of language barriers and unfamiliarity with the healthcare system. For the past 10 years, we have held a Japanese Community Outreach (JCO) Program for Japanese people in Pittsburgh to augment health literacy and to minimize health knowledge gaps along with encouraging networking among Japanese immigrants. We have held a weekend meeting each month at our University of Pittsburgh Medical Center (UPMC) Shadyside Family Health Center, providing childcare services and light refreshments, with support from UPMC Shadyside Hospital Foundation. When COVID‐19 started to hit neighboring states in March 2020, we decided to halt our monthly on‐site activities in order to minimize the risk of exposure to COVID‐19. At the same time, our clinic tried to minimize in‐person office visits along with maximizing virtual office visits. However, virtual office visits are supposed to be made through HIPPA compliant patient portal account, which requires patient social security numbers (SSN) and driver's license numbers in the United States. Unfortunately, these changes limited access to health care for some of our Japanese patients who do not have either SSN or the driver's license numbers. Additionally, most Japanese patients could not reach out to health professionals over the phone because of lack of English language skills. Also, because of the pandemic, they could not visit the clinic to schedule an appointment or communicate with our staff members as the alternative methods.

To address obstacles, we implemented three strategies. The first strategy was to switch the monthly meeting to a virtual meeting in April 2020. We emailed a Zoom meeting link to our list of members, and we also arranged telephone connections for elderly community. We covered virtual doctor's appointment, COVID‐19, obstetric care, self‐care with over‐the‐counter drugs, sleep, health insurance, and influenza. In addition to general information about COVID‐19, we discussed tips to cope with stressful situations during the pandemic, resources available in Japanese regarding domestic violence, and emergency contraception. More than 100 participants attended the meetings for the past 9 months. The second strategy was to provide an email address to enable Japanese patients to schedule appointments via email in Japanese. The third strategy was to share, via the JCO website, other useful and reliable Japanese website links to help patients access the latest information about COVID‐19 in the fluid setting.^1^ These strategies demonstrate the importance of acting quickly to maintain commutation with the Japanese community and address potential barriers to ongoing health care. We were able to use technology to compensate for limited ability to meet in person. These are great examples of transformation related to the COVID‐19 pandemic, providing helpful information to a disadvantaged minority in Japan.

## CONFLICT OF INTEREST

The author declares no conflict of interests for this article.
